# Quasi-3D Plasmonic Nanowell Array for Molecular Enrichment and SERS-Based Detection

**DOI:** 10.3390/nano10050939

**Published:** 2020-05-14

**Authors:** Sunho Kim, Chaewon Mun, Dae-Geun Choi, Ho Sang Jung, Dong-Ho Kim, Shin-Hyun Kim, Sung-Gyu Park

**Affiliations:** 1 Department of Chemical and Biomolecular Engineering, Korea Advanced Institute of Science and Technology, Daejeon 34141, Korea; shkim1020@kaist.ac.kr; 2 Advanced Nano-Surface Department (ANSD), Korea Institute of Materials Science (KIMS), Changwon, Gyeongnam 51508, Korea; apple1025@kims.re.kr (C.M.); jhs0626@kims.re.kr (H.S.J.); 3 Nano-Mechanical Systems Research Division, Korea Institute of Machinery & Materials (KIMM), Daejeon 305-343, Korea; lamcdg@kimm.re.kr

**Keywords:** nanoimprint lithography, 3D plasmonic nanowell array, surface-enhanced Raman spectroscopy, molecular concentration, signal uniformity

## Abstract

We report on a quasi-three-dimensional (3D) plasmonic nanowell array with high structural uniformity for molecular detection. The quasi-3D plasmonic nanowell array was composed of periodic hexagonal Au nanowells whose surface is densely covered with gold nanoparticles (Au NPs), separated by an ultrathin dielectric interlayer. The uniform array of the Au nanowells was fabricated by nanoimprint lithography and deposition of Au thin film. A self-assembled monolayer (SAM) of perfluorodecanethiol (PFDT) was coated on the Au surface, on which Au was further deposited. Interestingly, the PFDT-coated Au nanowells were fully covered with Au NPs with an ultra-high density of 375 μm^−2^ rather than a smooth film due to the anti-wetting property of the low-energy surface. The plasmonic nanogaps formed among the high-density Au NPs led to a strong near-field enhancement via coupled localized surface plasmon resonance and produced a uniform surface-enhanced Raman spectroscopy (SERS) response with a small relative standard deviation of 5.3%. Importantly, the highly uniform nanostructure, featured by the nanoimprint lithography and 3D growth of densely-packed Au NPs, minimizes the spatial variation of Raman intensity, potentially providing quantitative analysis. Moreover, analyte molecules were highly concentrated and selectively deposited in nanowells when a water droplet containing the analyte was evaporated on the plasmonic substrate. The analyte formed a relatively thick overcoat in the nanowells near the triple line due to the coffee-ring effects. Combining 3D plasmonic nanowell substrates with molecular enrichments, highly sensitive detection of lactic acid was demonstrated. Given its combination of high sensitivity and signal uniformity, the quasi-3D plasmonic nanowell substrate is expected to provide a superior molecular detection platform for biosensing applications.

## 1. Introduction

Plasmonic nanostructures provide strong light localization, through which the electromagnetic field is drastically enhanced in the nanogaps known as “hotspots”. This phenomenon has led to the development of an ultrasensitive molecular sensing method referred to as surface-enhanced Raman spectroscopy (SERS) [1,2,3,4,5,6,7,8,9]. The SERS detection technique has been used in medical diagnosis [10,11], environmental monitoring, and food safety control because it enables the sensitive, rapid, and label-free identification of molecules. The SERS intensity is highly sensitive to the arrangement, size, and topography of the plasmonic nanostructures. Therefore, the production of precisely controlled uniform metal nanostructures is very important to guarantee reliable molecular detection and quantitative analysis. Top-down approaches usually provide uniform and reproducible nanostructures in comparison with bottom-up approaches. The conventional top-down nanofabrication techniques to produce accurate structural arrangement and geometries for SERS applications include electron-beam lithography [12,13] and laser-interference lithography [14,15,16,17,18,19,20]. Although these techniques enable the elaborate construction of plasmonic nanostructures, the low-throughput and high-cost of production restrict practical uses. Furthermore, it is difficult to produce 3D nanostructures with the technique albeit the 3D geometries are highly desired to enhance sensitivity. Therefore, a robust and scalable fabrication method that simultaneously achieves high production-throughput, low cost, and high sensitivity of Raman measurement remains very important for commercializing prominent plasmonic technologies. 

Nanoimprint lithography (NIL) is a simple, reproducible, and scalable method to create plasmonic nanostructures over a large area so that it has been used for fabrication of SERS substrates [21,22,23,24,25]. The NIL enables the production of various periodic nanostructures on both rigid and flexible substrates. However, it is difficult to produce plasmonic nanostructures with densely packed sub-10 nm nanogaps for ultrasensitive sensing through the NIL itself. For example, Chou et al. [26] modified nanocompact disks with 10 nm features by NIL. To further enhance Raman intensity, on the other hand, it has been suggested to concentrate molecules in the nanogaps. Therefore, it is highly demanded, for the development of pragmatic sensing systems, to fabricate plasmonic nanostructures with a high density of nanogaps, through the NIL, that can concentrate and position probe molecules at the hotspots.

Here, we report on a 3D plasmonic hexagonal nanowell array with densely-packed Au nanoparticles (NPs) on the entire surface of Au nanowells, separated by a nanoscale-thick dielectric layer. The uniform hexagonal nanowell array with a well diameter of 400 nm and pitch of 500 nm was fabricated by NIL and a thin film of Au is deposited on the surface. The Au surface of the nanowell array was further decorated by a high density of Au NPs through the 3D growth of Au islands on 1*H*,1*H*,2*H*,2*H*-perfluorodecanethiol (PFDT)-coated, low-energy surface. The deposition condition of Au NPs was optimized through a finite-difference time-domain (FDTD) simulation and experiments to maximize the electric field enhancement and Raman signal intensity. The signal uniformity of the optimized plasmonic nanostructure was thoroughly evaluated through a microRaman mapping technique, which revealed that the signal intensity had a very small relative standard deviation of 5.3%. The nanowells can accommodate water and accumulate analyte molecules on their surface during the evaporation of a water droplet. In particular, analyte was highly concentrated to form a relatively thick overcoat on the Au NPs in nanowells near the triple line of the droplet. Combining 3D plasmonic nanowell substrates with molecular enrichments, highly sensitive detection of lactic acid, an excellent indicator of muscle tolerance, was demonstrated. The limit of detection (LOD) was measured to be 0.05 mM, which was 6 times lower than the LOD in the previous study [27]. 

## 2. Materials and Methods 

### 2.1. Fabrication of Gold Nanoparticle (Au-NP)-Decorated Nanowell Arrays 

A nanowell array in polymer substrates was doubly replicated from a Si master pattern with a hexagonal nanowell array through two steps of the imprinting process. Before the imprinting, the Si master mold was coated with an anti-adhesion molecule of hexamethyldisilazane (Sigma-Aldrich, Seoul, Korea) through vapor deposition to facilitate demolding. For both the first and second imprinting, an ultraviolet (UV)-curing resin (MINS-311RM, MINUTA Tech, Osan, Korea) were spin-coated onto a polyethylene terephthalate (PET) film for 30 s at a spin speed of 1000 rpm and the mold was pressed against the PET sheet under a pressure of 100 bar for 600 s using nanoimprinting equipment for a 5-inch wafer scale. After the double replication, a 100 nm-thick Au film was then directly deposited at the rate of 2.0 Å/s onto the polymer nanowell pattern using a sputtering system (SNTEK, Co. Ltd., Suwon, Korea). The Au nanowell substrate was then incubated for 2 h in a square dish to form a PFDT self-assembled monolayer (SAM). Afterward, another Au layer was deposited onto the PFDT-coated Au nanowell array at the rate of 0.3 Å/s using thermal evaporation (SNTEK, Co. Ltd., Suwon, Korea), which resulted in quasi-3D plasmonic nanowell substrates.

### 2.2. Characterization and Surface-Enhanced Raman Spectroscopy (SERS) Measurements

Surface morphologies were characterized by field-emission scanning electron microscopy (FE-SEM; JSM-6700F, Jeol, Tokyo, Japan). The SERS spectra of methylene blue (MB) were recorded using a handheld Raman spectrometer (CBEx, Snowy Range Instrument, Laramie, WY, USA) with a laser wavelength of 633 nm and 785 nm, and laser power of 10 mW. A 3 μL of 5 × 10^−6^ M MB solution was dropped on the surface of the 3D SERS substrate and the solvent was evaporated. For microRaman mapping, the 3D plasmonic nanowell substrate was dipped into 1 mM benzenethiol (BT) solution for 24 h for complete surface coverage of probe molecules and the substrate was then washed with ethanol several times and dried gently under N_2_ gas. The microRaman mapping spectra were obtained using a high-resolution dispersive Raman microscope (Horiba Jobin Yvon, LabRAM HR, Kyoto, Japan) with 50× objective lens (NA = 0.75) and a 632.8 nm HeNe laser. A 16 µm × 16 µm region was measured at 1 µm resolution with a laser power of 0.4 mW. 

### 2.3. Numerical Simulations

Electromagnetic simulations of quasi-3D plasmonic nanowell structures were performed with the commercial finite-difference time-domain (FDTD) software package (version 8.21, Lumerical Solutions, Seoul, Korea). The nanostructure was sketched based on scanning electron microscope (SEM) observations using Autodesk Fusion 360, and the data were subsequently exported into FDTD software. A linearly polarized plane wave with a wavelength of 633 nm was incident to the Au nanowell. Because the Au film on the UV-resin surface was 100 nm thick, we assumed that no light was transmitted through it. The dielectric constant of Au was set to *ε*_Au_ = −8.962364 + 1.164*i* at 633 nm, and the refractive index of PFDT was set to 1.333 [2]. 

## 3. Results and Discussion

**Figure 1:**
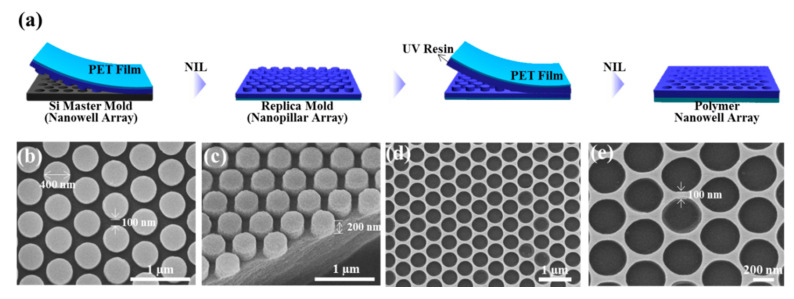
Fabrication of nanowell array. (**a**) Schematic of the fabrication process of the polymer hexagonal nanowell array using nanoimprint lithography (NIL). (**b**,**c**) Scanning electron microscopy (SEM) images showing the top view and 45°-tilted view of a replica mold with a nanopillar array. (**d**,**e**) SEM images of polymer nanowell array replicated from the nanopillar soft mold.

A schematic for the fabrication process of the nanowell array is shown in Figure 1a. A Si master pattern with a hexagonal nanowell array was transferred to a resin-coated PET film; the array has a well diameter of 400 nm, a height of 200 nm, and a pitch of 500 nm. The replicated structure on the PET film had a nanopillar array with the same dimensions as the master pattern (Figure 1b,c). The nanopillar array was replicated again to make a nanowell array with the same structure as the original master mold. The resulting nanohole array is highly uniform (Figure 1d,e). The polymer nanowell pattern was used for making plasmonic nanowell array with densely-packed Au NPs. The quasi-3D nanowell structures prepared using NIL have several advantages for SERS-based sensing applications: (1) the NIL process is cost-effective and the nanostructures are reproducible; (2) the resulting plasmonic nanostructures exhibit high signal uniformity; and (3) molecules can be accumulated in the plasmonic nanowells to further enhance Raman intensity.

The quasi-3D periodic nanowell structures were prepared by following three steps with the NIL-featured nanowell array. First, a 100 nm-thick Au layer was deposited onto the polymer nanowell array. Second, PFDT SAM was deposited on the Au surface through a vapor-phase deposition. The thiol (–SH) groups of PFDT compactly formed a monolayer at atmospheric pressure because of their strong affinity with the Au layer. The fluoro (–F) groups render the PFDT-coated Au surface to have very low surface energy [5,28]. Third, another Au layer was deposited onto the PFDT-coated Au nanowell array. 

**Figure 2:**
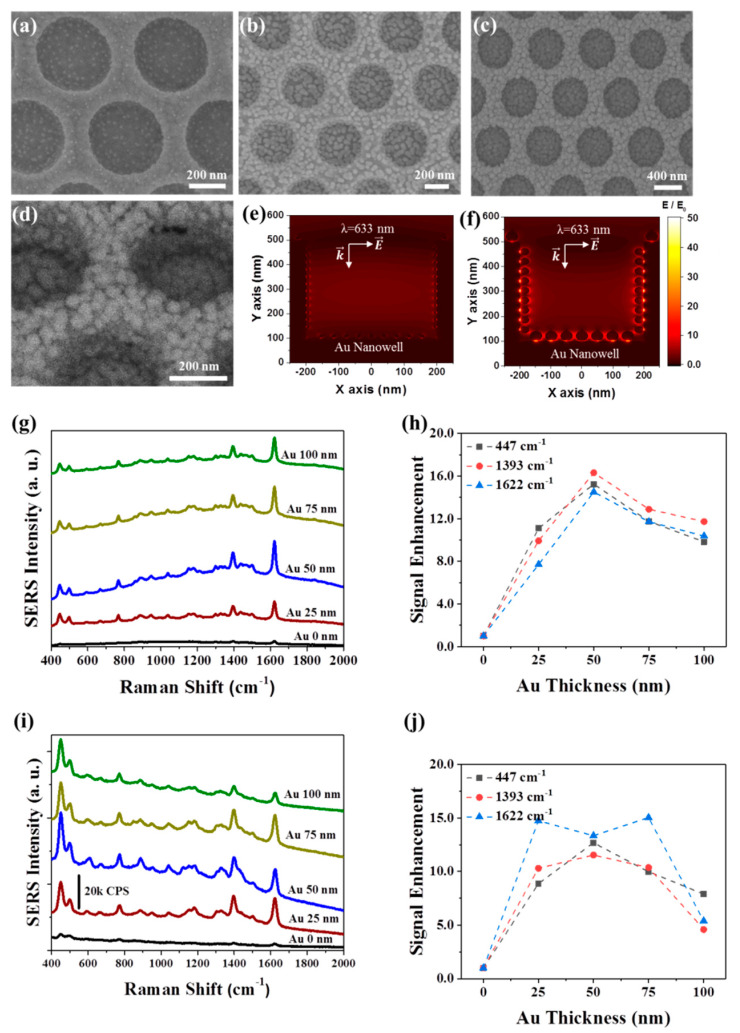
Optimization of Au deposition condition. (**a**–**d**) SEM images of the quasi-3D Au nanowell structures with four different Au deposition conditions, where the conditions for the formation of Au films with thicknesses of (**a**) 5 nm, (**b**) 25 nm, and (**c**,**d**) 50 nm on normal surfaces are used. (**e**,**f**) Spatial distributions of electric field intensity in quasi-3D Au nanowells for the conditions of (**e**) 5 nm-thick and (**f**) 50 nm-thick Au film formation. The diameters of Au NPs on the sidewall and bottom surfaces and a gap spacing are set to 10 nm, 14 nm, and 24 nm for the condition of 5 nm-thick Au film and those are set to 30 nm, 40 nm, and 9 nm for the condition of 50 nm-thick Au film, based on SEM observation. Raman spectra of methylene blue (MB) taken using the quasi-3D Au nanowells with 5 different deposition conditions of Au deposition using (**g**) 633 nm and (**i**) 785 nm lasers. Variation of surface-enhanced Raman spectroscopy (SERS) intensity at Raman shifts of 447 cm^−1^, 1393 cm^−1^, and 1622 cm^−1^ according to the Au deposition condition for (**h**) 633 nm and (**j**) 785 nm lasers.

Figure 2a shows the nanowell array after the deposition of Au onto PFDT-coated Au nanowells, where the condition for the formation of a 5-nm-thick Au layer on normal surfaces by conformal deposition was used. Each nanowell contains Au NPs that are distributed on the entire surfaces. The high surface energy of Au (*γ*_Au_ = 1.54 J m^−2^) and the low surface energy of PFDT (*γ*_PFDT_ = 0.015 J m^−2^) [29] may cause negative spreading parameter, which results in the formation of spherical Au NPs rather than a smooth film on the surface of the PFDT-coated Au. This anti-wetting property of the PFDT SAM caused the formation of Au NPs at 3D growth mode [30] and enabled the formation of dense nanogaps among Au NPs by increasing the size of Au NPs. As the dose of Au deposition increases, so does the size of the discrete Au NPs, resulting in a smaller gap distance (i.e., nanogap) among the Au NPs (Figure 2b). When the condition for the 50-nm-thick Au layer on normal surfaces is used, a dense array of sub-50 nm Au NPs was formed on the entire quasi-3D surfaces of the bottom surface and sidewall of the nanowells and interstitial area among the nanowells (Figure 2c,d). All nanowells showed a high structural uniformity. 

We investigated the near-field coupling of the quasi-3D plasmonic nanowell structures using FDTD simulations. For two different deposition conditions of 5 nm-thick and 50 nm-thick Au layers on normal surfaces, the size and spacing of Au NPs were investigated by SEM observation, which were used for the construction of model structures for FDTD simulations; the Au NPs formed on the bottom surface is relatively larger than that on the sidewall of nanowells due to the directional flux of Au atoms during thermal evaporation: Average diameters of Au NPs on the sidewall and bottom surface were 10 nm and 14 nm for the condition of a 5 nm-thick Au layer and 30 nm and 40 nm for the condition of 50 nm-thick Au layer, respectively. For plane-wave radiation with a wavelength of 633 nm, the electric field was strongly localized in the gaps between the Au NPs and Au film (Figure 2e,f). Interestingly, the gaps on the sidewall show the high near-field enhancement because the gap is aligned parallel to the polarization direction of the incident light. The electric field is one-order of magnitude larger for the condition of the 50 nm-thick Au layer than the condition of the 5 nm-thick Au layer. 

Next, we optimized the condition of Au deposition to maximize SERS performance. For this purpose, we used an organic dye, methylene blue (MB), as a probe molecule. A 3 μL of 5 × 10^−6^ M MB aqueous solution was dropped on the surface of the quasi-3D SERS substrate, and the solvent was evaporated. The SERS signal was then measured using an excitation wavelength of 633 nm and 785 nm handheld Raman spectrometers. The quasi-3D nanowell substrates prepared using 50 nm-thick Au film conditions showed the highly enhanced SERS signal for both laser wavelengths (Figure 2g–j). This was due to the fact that the quasi-3D nanowell substrates with 50 nm thick Au film conditions had the highest nanogap density of 375 μm^−2^, but the nanogap density decreased for the conditions of 75 nm-thick and 100 nm-thick Au films due to fusion of Au NPs, as shown in Figure S1. When the condition for 25 nm-, 50 nm-, and 75 nm-thick Au layer on normal surfaces was used, the average diameters of Au NPs were 33 nm, 43 nm, and 54 nm, respectively.

**Figure 3:**
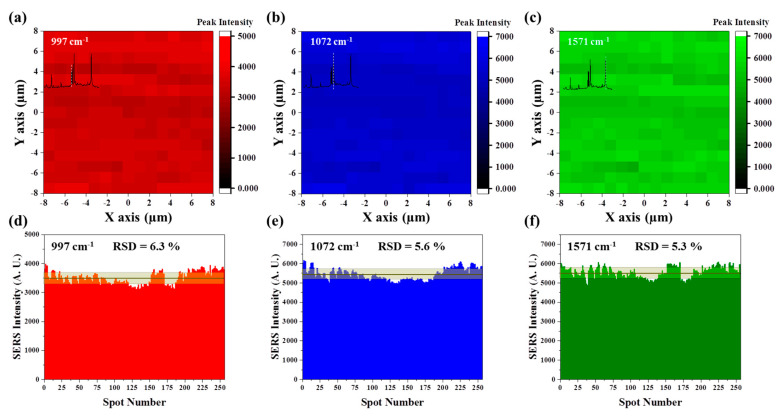
High uniformity of Raman intensity. (**a**–**c**) Spatial distribution of Raman intensities of benzene thiol (BT) at characteristic peak positions of (**a**) 997 cm^−1^, (**b**) 1072 cm^−1^, and (**c**) 1571 cm^−1^ acquired by micromapping. (**d**–**f**) Variation of Raman intensities on 256 spots. The relative standard deviation (RSD) of the intensity is denoted in each panel.

Signal uniformity and reproducibility were very important to guarantee the quantitative analysis. As the quasi-3D nanowells had minimal structural variation nanowell-by-nanowell as confirmed in Figure 2, it was expected that they would show high signal uniformity and reproducibility. To investigate this, the substrate was subjected to Raman micromapping (Figure 3). To avoid any variation of molecular concentration and study the influence of structural effect alone, the quasi-3D nanowell substrate was dipped into a 1 mM benzenethiol (BT) solution for 24 h rather than dropping and drying the solution on the substrate; the dipping made a complete monolayer coating of BT on Au surface. The area of 16 μm (*x*-axis) × 16 μm (*y*-axis) on the BT-coated substrate was scanned by a resolution of 1 μm × 1 μm. Consequently, a total of 256 pixels (1 pixel = 1 μm × 1 μm) were obtained and the characteristic peaks of BT at 997, 1072, and 1571 cm^−1^ were used to assess the signal uniformity. Highly uniform distribution of Raman intensities was observed for the quasi-3D nanowell substrate for all three peak positions (Figure 3a–c). According to our statistical analysis, the relative standard deviation (RSD) for the three different peaks was as small as 6.3% for 997 cm^−1^ (Figure 3d), 5.6% for 1072 cm^−1^ (Figure 3e), and 5.3% for 1571 cm^−1^ (Figure 3f). This high signal uniformity is contributed from the minimal structural variation on the substrate as the quasi-3D nanowells were featured by highly-reproducible NIL and growth of high density Au NPs on the low-energy surface. 

**Figure 4:**
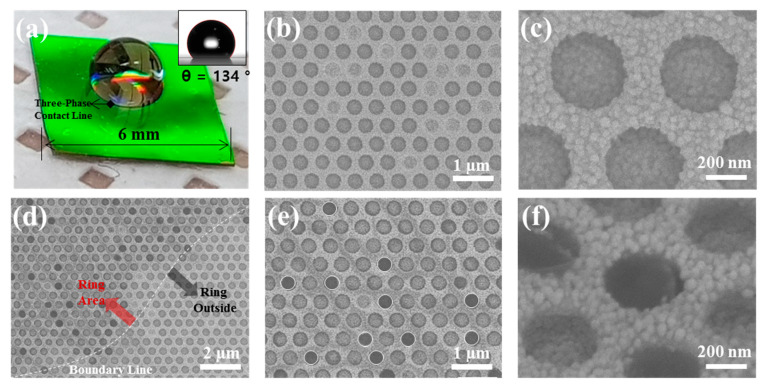
Molecular enrichment and selective deposition in nanowells. (**a**) Photograph of a water droplet on the quasi-3D plasmonic nanowell substrate. The inset shows a profile of the droplet with a contact angle of 134°. (**b**–**f**) SEM images of the plasmonic nanowell array taken after complete evaporation of water droplet containing MB, where the images were taken at the central area of the substrate (**b**,**c**), the boundary between the ring-shaped stain and outside (**d**), and the ring (**e**,**f**). The white circles in (**e**) indicate dark nanowells that contain a relatively thick MB overcoat. The SEM image of (**f**) is a 45°-tilted view of the nanowell containing the thick MB overcoat.

Because the quasi-3D nanowell structures contain a fluorine-based PFDT interlayer, the surface was hydrophobic. We characterized the hydrophobic properties by dropping an aqueous solution onto the structures, as shown in Figure 4a; the periodic nanowell array developed the vivid green color through wavelength-selective diffraction [31]. The water droplet showed a contact angle (CA) of 134° (the inset of Figure 4a). When the droplet contained MB, the evaporation of water concentrated MB and left behind a ring-shaped stain due to coffee-ring effect [32]. All nanowells on the central area and rings contained MB on their surface. In the central area, MB was deposited on the inner surfaces of the nanowells rather than the interstitial surface among the nanowells (Figure 4b,c); the darker parts in the SEM images have the overcoat of dielectric MB molecules on the Au NPs, whereas bright parts are the Au NPs without the overcoat. This selective deposition in the nanowell is attributed to the trapping of water in the nanowell at the last moment of evaporation due to the transition of wetting state from Cassie-Baxter to Wenzel [2]. The amount of MB deposited in nanowells in the ring-shaped stain was larger than those in the central area, as expected from the coffee-ring effect (Figure 4d–f). Many nanowells appear much darker than those in the central area, which was caused by the formation of a relatively thick MB overcoat in the nanowells (denoted with white circles in Figure 4e). The molecular enrichment and selective deposition in nanowells potentially provide highly sensitive molecular detection for water-soluble molecules.

**Figure 5:**
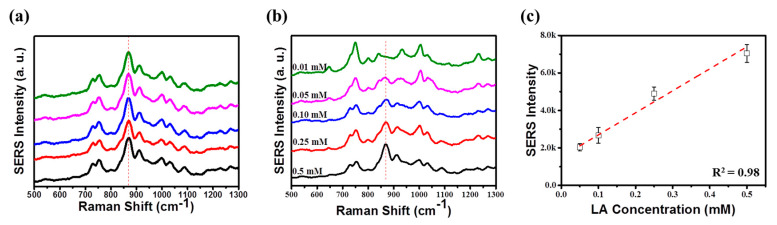
(**a**) Raman spectra of 0.5 mM lactic acid (LA) measured from the five random spots of quasi-3D nanowell structures. The characteristic peak of lactic acid at 869 cm^−1^ peak was used to assess the signal uniformity. The RSD was as small as 6.8% at 869 cm^−1^. (**b**) Comparison of the Raman spectra of lactic acid obtained from 0.5 mM to 0.01 mM lactic acid-treated 3D plasmonic nanostructures. (**c**) Calibration plot with four known concentrations. The SERS intensity at 869 cm^−1^ was used for the calibration plot.

As proof-of-concept, we used the optimized plasmonic chips to detect lactic acid. Lactic acid accumulates between cells unless the tissue is supplied with enough oxygen to support aerobic oxidation of glucose. Because the concentration of lactic acid in the blood is related to the rate of muscle cell production and liver metabolism, lactic acid has been widely used as an indicator of muscle tolerance [27,33]. A 3 μL of lactic acid aqueous solution with six different concentrations, from 0.5 to 0.01 mM were dropped on the surface of the quasi-3D SERS substrate, and then the solvent was evaporated. The Raman spectra were recorded by measuring five random spots of quasi-3D nanowell structures for 0.5 mM concentration, using a 633 nm portable Raman spectrometer (Figure 5a). The characteristic peak of lactic acid at 869 cm^−1^ peak was used to assess the signal uniformity [27]. The RSD was as small as 6.8% for 869 cm^−1^. Once again, we confirmed the high signal uniformity of the 3D plasmonic nanostructures. Figure 5b shows quantitative analysis of lactic acid on 3D plasmonic nanostructures. The SERS intensity of Raman band at 869 cm^−1^ changes proportionally with lactic acid concentration. For 0.01 mM, no characteristic peaks are detected and only background signal from dielectric interlayer is detected [5], indicating that the LOD is measured to be 0.05 mM, which is 6 times lower than the LOD in the previous study [27]. A calibration plot with four known concentrations ranging from 0.5 mM to 0.05 mM was obtained from with a good correlation coefficient of 0.98 (Figure 5c). 

## 4. Conclusions

In this work, we fabricated quasi-3D plasmonic nanowell structures by combining highly reproducible, scalable, and cost-effective NIL and 3D growth of Au NPs on an ultra-low-energy surface. Au NPs grown on the sidewall and bottom surface of nanowell formed strong hotspots at the nanogaps with the Au-coated surfaces and interstitial voids, amplifying Raman signal for the molecules deposited on the hotspots and thereby providing highly sensitive molecular detection. Moreover, the quasi-3D nanowell array exhibited a very uniform Raman signal, with a relative standard deviation as small as 5.3% in microscopic mapping analysis, as the quasi-3D plasmonic nanostructures were uniformly created with a negligible structural variation. An analyte can be concentrated and selectively deposited in nanowells by drying an analyte-dissolved droplet, which potentially improves Raman sensitivity. We believe that the combination of high sensitivity and signal uniformity as well as reproducible and low-cost production will provide a new opportunity for translating prominent plasmonic technologies toward commercialization, especially in the area of biosensors. 

## Supplementary Materials

The following are available online at https://www.mdpi.com/2079-4991/10/5/939/s1, Figure S1: SEM images of the Au nanowells whose surfaces are decorated with Au NPs using deposition thickness of (a) 25 nm, (b) 50 nm, and (c) 75 nm. (d) Histogram of the size distributions of spherical Au NPs decorated on Au nanowells for the three different deposition conditions, as denoted. The solid lines are fitting curves with the Gaussian function.
