# Supplementary material for: Quasi-3D Plasmonic Nanowell Array for Molecular Enrichment and SERS-Based Detection
Source: Nanomaterials (Basel). 2020 May 14;10(5):939. doi: 10.3390/nano10050939 (PMC7279529; doi:10.3390/nano10050939)
Supplement: Supplementary file 1 [file nanomaterials-10-00939-s001.pdf]

Supplementary Materials

Quasi-3D Plasmonic Nanowell Array for Molecular Enrichment and SERS-Based Detection

Sunho Kim [1], Chaewon Mun [2], Dae-Geun Choi [3], Ho Sang Jung [2], Dong-Ho Kim [2,*], Shin-Hyun Kim [1,*] and Sung-Gyu Park [2,*]

[1] Department of Chemical and Biomolecular Engineering, Korea Advanced Institute of Science and Technology, Daejeon 34141, Korea; shkim1020@kaist.ac.kr (S.K.)

[2] Advanced Nano-Surface Department (ANSD), Korea Institute of Materials Science (KIMS), Gyeongnam 51508, Korea; apple1025@kims.re.kr (C.M.); jhs0626@kims.re.kr (H.S.J.)

[3] Nano-Mechanical Systems Research Division, Korea Institute of Machinery & Materials (KIMM), Daejeon 305-343, Korea; lamcdg@kimm.re.kr (D.-G.C.)

* Correspondence: sgpark@kims.re.kr (S.-G.P.); kim.sh@kaist.ac.kr (S.-H.K.); dhkim2@kims.re.kr (D.-H.K.) Tel.: +82-55-280-3632 (S.-G.P.)

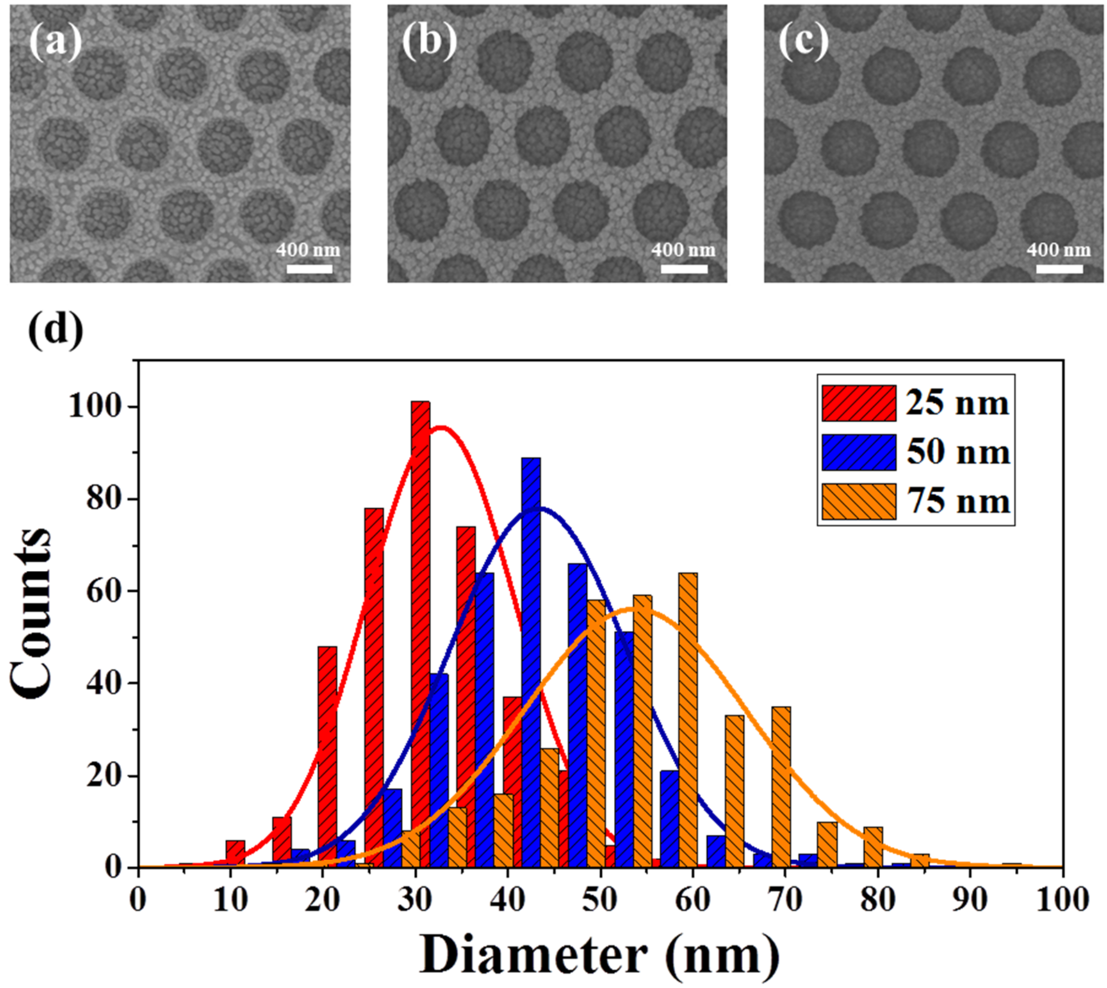

Figure S1. SEM images of the Au nanowells whose surfaces are decorated with Au NPs using deposition thickness of (**a**) 25 nm, (**b**) 50 nm, and (**c**) 75 nm. (**d**) Histogram of the size distributions of spherical Au NPs decorated on Au nanowells for the three different deposition conditions, as denoted. The solid lines are fitting curves with the Gaussian function.
